# Remarkably enhanced performance of the metathesis reaction of ethylene and 1-butene to propene using one-step prepared W-MCM-41 catalysts

**DOI:** 10.1039/c9ra08646c

**Published:** 2019-12-09

**Authors:** Changji Wang, Feng Jiang, Guangzheng Zuo, Bing Liu, Hanxu Li, Xiaohao Liu

**Affiliations:** College of Earth and Environment, Anhui University of Science and Technology 232001 Huainan China; Department of Chemical Engineering, School of Chemical and Material Engineering, Jiangnan University Wuxi 214122 China liuxh@jiangnan.edu.cn

## Abstract

Highly dispersed tungsten species with an isolated tetrahedral WO_*x*_ species structure are substantially beneficial for the metathesis reaction of ethylene and 1-butene to propene. The conventional impregnation method always leads to the formation of inactive crystalline WO_3_ thereby notably decreasing the amount of active sites. In this study, we synthesized a highly dispersed W-MCM-41 catalyst using the one-step precipitation method with a Si/W ratio of 30. The prepared catalyst showed excellent catalytic performance with a 1-butene conversion of 92.7% and a propene selectivity of 80.8%. In contrast, the impregnated catalyst with the same W loading as the one-step precipitation method resulted in a much lower 1-butene conversion of 76.5% and propene selectivity of 34.1%. Various characterization techniques including XRD, XPS, ICP-OES, UV-vis DRS, TEM, and Raman spectroscopy were applied to confirm that the one-step precipitation method can efficiently prepare well-dispersed W-MCM-41 catalysts with the desired structure in spite of the fact that the ideal dispersive structure was strongly dependent of the Si/W ratio and stirring time of the reaction mixture of tungstic acid and TEOS. In addition, the introduction of an upstream catalyst onto the W-MCM-41 catalyst could not obviously improve the 1-butene conversion and propene selectivity, which might be due to fast 1-butene isomerization easily occurring on the abundant Si–OH of the W-MCM-41 catalyst. This work provides new insights for the design of metathesis catalysts and reaction processes to efficiently convert ethylene and 1-butene into propene.

## Abstract 2

The W-MCM-41 catalysts synthesized *via* one-step precipitation method showed the excellent activity and selectivity for the metathesis reaction of ethylene and 1-butene to propene.

## Graphical abstract

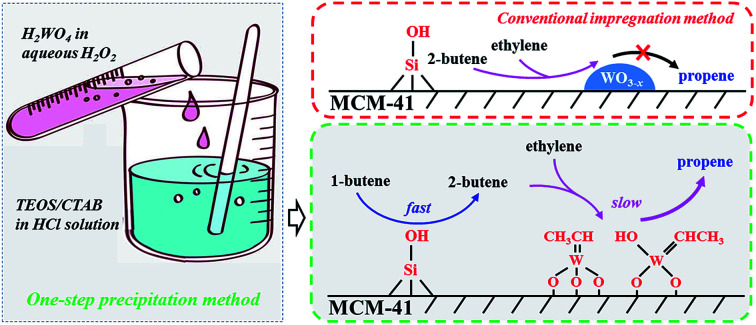

## 1. Introduction

Propene is one of the most important building blocks in the chemical industry. The demand for propene has been continuously increasing at an average rate of 4–5% per year.^1–7^ The Fischer–Tropsch to olefins (FTO) process to produce light olefins has attracted considerable interest because of the availability of coal-, natural gas-, and biomass-derived syngas (CO + H_2_).^8,9^ However, the FTO route usually generates different kinds of light olefins, including ethylene, propene, and 1-butene.^10,11^ Despite the high selectivity to total light olefins, the selectivity to propene is always lower than 40%. Therefore, it is necessary to develop an efficient approach to enhance the propene fraction and simultaneously suppress the generation of ethylene or 1-butene. Olefin metathesis is a highly versatile process for interconverting olefinic hydrocarbons.^12^ Propene can be formed through the metathesis reaction of ethylene and 1-butene.^13–15^ Therefore, the metathesis reaction of ethylene and 1-butene to propene is a promising method to increase the selectivity toward propene from FTS products.

Supported WO_3_ catalysts exhibit better activity, excellent stability, anti-poisoning property, and low price and thus are highly desirable for industrial application.^16–21^ It is widely accepted that the highly dispersed WO_*x*_ species and its existing state on supports play critical roles in the catalytic performance.^22^ The properties of supports and the preparation methods have a significant effect on the structure and dispersion of WO_*x*_ species, and thus significantly influence the catalytic performance.^23^ Mesoporous materials, such as HMS, SBA-15, MCM-41, and FDU-12, have uniform pores, large BET surface areas and pore volumes, which are beneficial for the dispersion of active species and the transportation of reactants and products.^24–29^ Thus, mesoporous materials have been considered as promising catalyst supports in olefins metathesis.

In our previous study, we investigated the structure of tungsten oxide supported on SBA-15 and its catalytic performance in the metathesis reaction of 1-butene and ethylene to propene.^30^ It was found that the propene production through the metathesis reaction consists of three steps, including 1-butene isomerization, W–carbene formation, and metathesis reaction. The silanol group (Si–OH) in SBA-15 is acting as the active site for 1-butene isomerization. The W–carbene (W=CH–CH_3_) species are formed *via* the partial reduction of isolated tetrahedral WO_*x*_ species which contain W=O or W–OH bonds. The W–carbene species play a crucial role in the metathesis reaction. Furthermore, the W/SBA-15 pretreated by H_2_O leads to a decrease of the metathesis activity. This is mainly attributed to the sintering of isolated WO_*x*_ species, forming an inactive crystalline WO_3_ phase. These results suggest that the nature of the supported tungsten oxide phase significantly affects the catalytic performance of the olefin metathesis reaction. Establishing the structure–activity/selectivity relationships for metathesis reaction would be helpful for the rational design of more efficient catalysts.

In present investigation, MCM-41 molecular sieve with two-dimensional pore structure, smaller pore size and larger surface area was used as the catalyst support to study the olefin metathesis reaction. The WO_*x*_ species were anchored onto the framework of MCM-41 by one-step precipitation method, which can improve the dispersion of tungsten active species and thus might have a significant impact on the catalytic activity and selectivity. The effects of stirring time and Si/W ratio on the catalyst structures and corresponding reaction performances were systematically investigated. Moreover, the effect of upstream isomerization catalyst on the improvement in the catalytic activity and selectivity was also studied.

## 2. Experimental methods

### 2.1 Catalyst preparation

The W-MCM-41(*z*) catalyst was prepared by a one-step precipitation method,^31^ where *z* represents the Si/W molar ratio. Taking W-MCM-41(50) as an example, the typical preparation procedure was as follows: (1) 0.625 g (2.5 mmol) of tungstic acid (H_2_WO_4_) was added into 5 mL of 15 wt% aqueous H_2_O_2_, and then the mixed solution was stirred at 60 °C for 1 h to generate soluble low-condensed peroxo species. After that, the solution was cooled to room temperature and centrifuged to remove undissolved tungstic acid. The resulted solution was labelled as S1. (2) 5.467 g (15 mmol) CTAB powder, 240 mL deionized water and 92 mL 12.5 mol L^−1^ hydrochloric acid solution were mixed to obtain a transparent solution. 28 mL (0.125 mol) of TEOS was mixed with the obtained solution, and solution S1 was immediately added, gradually forming the precipitate. (3) The obtained reaction mixture was stirred at room temperature for 5 h (defined as the stirring time and various stirring time designed to investigate its effect on the catalyst structure), and then the mixture underwent vacuum filtration and washing by deionized water until the pH reached neutral, obtaining a light yellow solid which was then dried overnight at 40 °C. (4) The dried solid was calcined in air at 650 °C for 4 h with a heating rate of 1 °C min^−1^ to obtain W-MCM-41(50) catalyst powder.

As a comparison of the one-step prepared W-MCM-41(30) catalyst, the impregnated 18W/MCM-41 is also prepared with the same loading of W in the two catalysts. The catalyst support, MCM-41, is prepared as the above W-MCM-41(*z*) catalyst without the addition of W precursor. The W species was impregnated into the MCM-41 support with the W loading of 18 wt% by an incipient wetness impregnation method as our previous work.^30^

### 2.2 Catalyst characterization

Brunauer–Emmett–Teller (BET) surface area, pore volume, pore size, and the adsorption–desorption isotherms were measured by using a Micromeritics ASAP 2020. The X-ray diffraction (XRD) patterns of samples were recorded on a Bruker AXS D8 Advance X-ray diffractometer using Cu (Kα) radiation with 0.02° step in the 2*θ* range from 10 to 80°. Diffuse-reflectance UV-vis spectra (UV-vis DRS) were obtained in the range of 200–700 nm with a Shimadzu UV-3600 Plus Spectrometer, using BaSO_4_ as reference. The Raman spectra of the samples were recorded on a Renishaw Micro Raman Spectrometer in the range from 200 to 1200 cm^−1^ (excitation line: 785 nm of diode solid-state laser). Transmission electron microscopy (TEM) images of the catalysts were taken in a Tecnai G2 F30 S-Twin TEM operating at 200 kV. The XPS data were collected using X-ray photoelectron spectrometer Thermo SCIENTIFIC ESCALAB 250xi with an Al-Kα (1486.8 eV) X-ray source. ICP-OES were performed on an Agilent 5100 inductively coupled plasma-optical emission spectrometer.

### 2.3 Catalytic evaluation

The catalytic experiments of W-based catalysts for the metathesis reaction of ethylene and 1-butene to propene were carried out in a fixed bed reactor. 0.5 g of shaped catalyst (40–60 mesh) was placed at the center of reactor. The W-based catalysts need to be pretreated at 550 °C and 1 bar for 4 h in pure N_2_. The flow rate of pure N_2_ was 35 mL min^−1^. After pretreatment and cooling to designed reaction temperature, for example 450 °C, the mixed feed gas (ethylene/1-butene = 2/1 in molar ratio) was fed into the catalyst bed. The gas weight hourly space velocity (WHSV), defined as the weight ratio of mixed gas to catalyst packed, was regulated at 1.8 h^−1^. Two mass flow controllers (MFC, Brooks, model 5800E) controlled the flow rates of ethylene and 1-butene, respectively. All the products were analyzed online by a gas chromatograph (Agilent, GC7820A) with a HP-plot/Al_2_O_3_ KCl column using a flame ionization detector (FID). The 1-butene conversion and products selectivity were calculated according to the equations reported in our previous study.^30^

## 3. Results and discussion

### 3.1 Influence of stirring time on the catalytic performances of W-MCM-41 catalyst

**Fig. 1:**
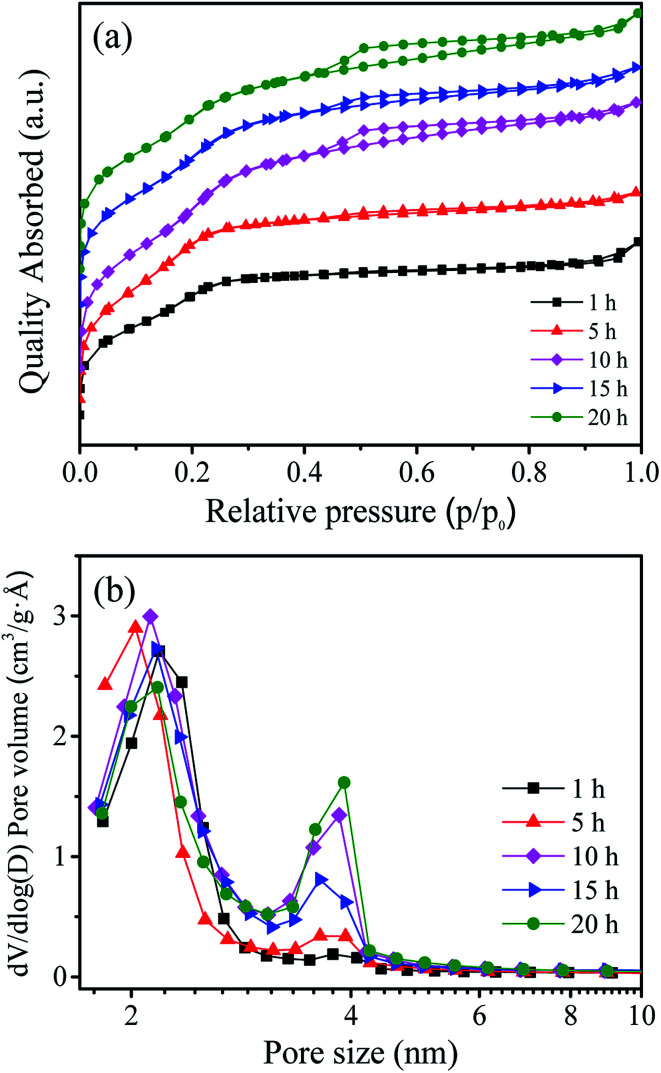
N_2_ adsorption–desorption isotherms (a) and pore size distribution (b) of the W-MCM-41(50) catalysts with different stirring time. The isotherms have been moved verticality to avoid the overlap of isotherms.

Table 1 shows the BET surface area, pore volume and pore size of the as prepared W-MCM-41 catalysts with different stirring time. It can be seen that the BET surface area firstly increases and then decreases as the increase of stirring time from 1 to 20 h, and the sample obtained with 10 h stirring time shows both the largest BET surface area of 970 m^2^ g^−1^ and the largest pore volume of 0.61 m^3^ g^−1^. As shown in Fig. 1a, the prepared catalysts are typical mesoporous materials which display obvious type IV isotherms with very clear H_1_-hysteresis loops as the increase of stirring time up to 5 h.^32^ As indicated by the pore size distribution of these samples in Fig. 1b, the pore size is mainly at about 2 nm when the stirring time is below 5 h. With an increase in the stirring time, some larger pores of about 4 nm appear and the amount of these larger pores increases. In general, the surface area, pore size and pore volume are obviously influenced by the stirring time.

**Table 1:** Textural properties of W-MCM-41(50) catalyst with different stirring time

Stirring time (h)	BET surface area (m^2^ g^−1^)	Pore volume^a^ (cm^3^ g^−1^)	Pore size^a^ (nm)
1	703	0.44	2.60
5	879	0.44	2.38
10	970	0.61	2.60
15	860	0.53	2.62
20	847	0.57	2.80

a Evaluated by the BJH method.

**Fig. 2:**
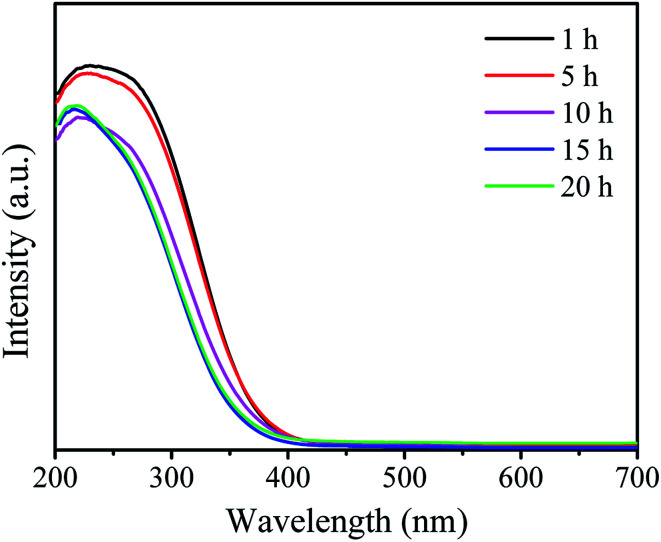
UV-vis DRS spectra of the W-MCM-41(50) catalysts with different stirring time.

The UV-vis DR spectra can provide reliable information about the local molecular coordination and bonding of W species. As shown in Fig. 2, all the samples show a typical peak at ∼220 nm and a weaker peak at ∼270 nm indicating the presence of the isolated tetrahedral structure and the oligomeric octahedral structure of WO_*x*_ species, respectively.^33^ Moreover, the peak at ∼400 nm assigned to the d–d band of crystalline WO_3_ is not observed in all the samples.^31^ These results suggest that the WO_*x*_ species are highly dispersed on the surface of support and no crystalline WO_3_ is formed.

**Fig. 3:**
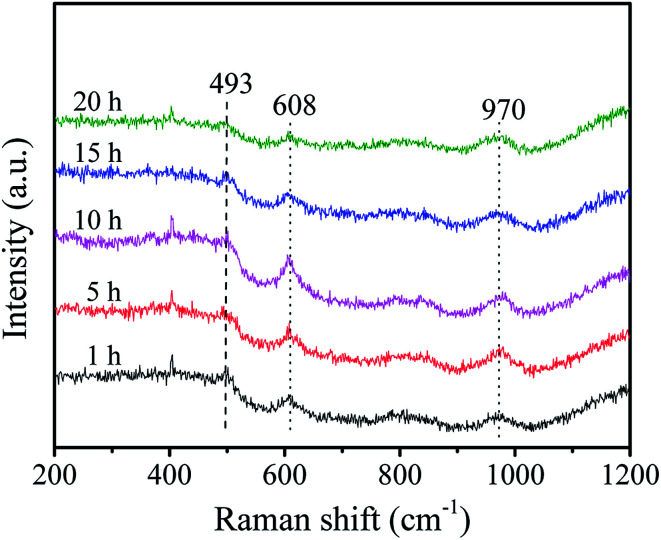
Raman spectra of the W-MCM-41(50) catalysts with different stirring time.

Raman spectra were further carried out to reveal the structural properties of these samples. As shown in Fig. 3, the vibrational bands at 493 and 608 cm^−1^ are attributed to the D2 and D1 defect modes related to tri- and tetra-cyclosiloxane rings of the support, respectively.^34^ The 970 cm^−1^ Raman band arises from the terminal *vs.* (W=O) of surface WO_*x*_.^35^ The band intensity of support firstly decreases and then increases as the increase of stirring time and the maximum intensity is obtained with stirring time of 10 h. Combined with the BET results, it's deduced that the good crystallinity of the support favors the high surface area. There is slight influence of stirring time on the peak intensity of W=O bond in the sample as the stirring time of 5 h shows a slight higher peak which indicates the presence of more surface WO_*x*_ species.

**Fig. 4:**
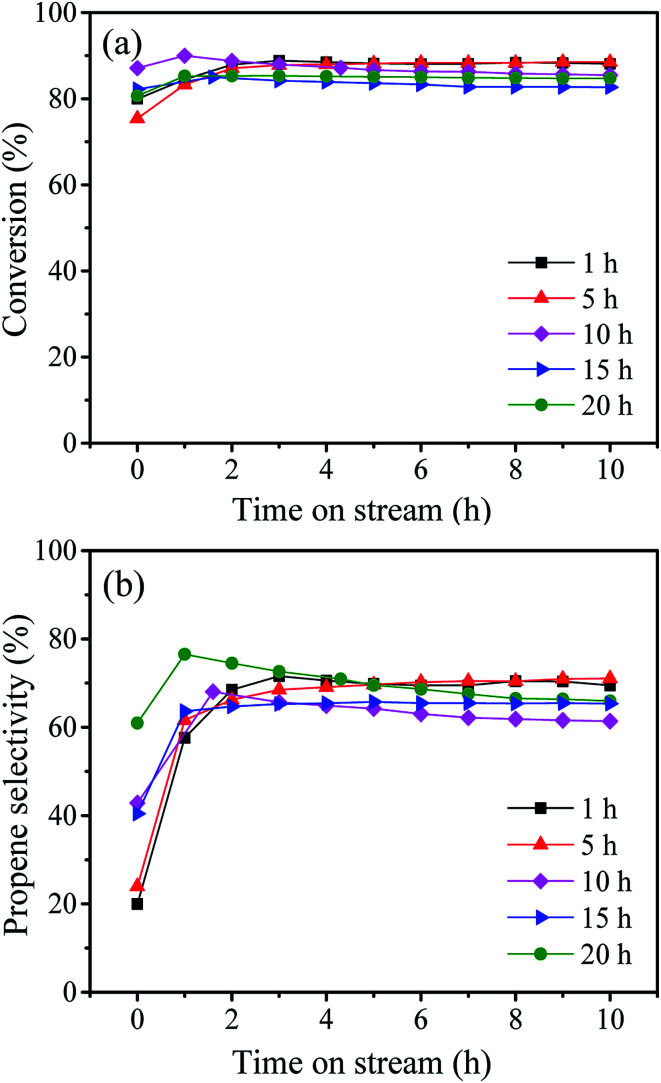
Time-dependence of the 1-butene conversion (a) and propene selectivity (b) over W-MCM-41(50) catalyst with different stirring time.

These samples were tested for the metathesis reaction of 1-butene and ethylene to propene and the results are shown in Fig. 4 and Table 2. It can be seen from Fig. 4a that all the catalysts show stable 1-butene conversion after reaction for 3 h except the cases with stirring time longer over 10 h which show slight deactivation with time on stream. As shown in Fig. 1b, some larger pores at about 4 nm are formed at a longer stirring time over 10 h. The catalyst with stirring time of 5 h shows the highest 1-butene conversion of 88.3% and propene selectivity of 70.4% (Table 2). The higher conversion and selectivity over the catalyst with stirring time of 5 h is assigned to the higher amount of WO_*x*_ species with the isolated tetrahedral structure and W=O bond (Fig. 2 and 3). The detailed relationship between catalyst structure and catalytic performances will be discussed later. Thus, a suitable stirring time should be selected to prepare the excellent metathesis catalyst for the efficient conversion of ethylene and 1-butene to propene. In the following part, the stirring time is assigned to 5 h for all the prepared W-MCM-41 catalysts.

**Table 2:** Catalytic performances of W-MCM-41(50) catalyst with different stirring time^a^

Stirring time (h)	Conversion (%)	Selectivity (%)				
		Propene	C_5+_	*trans*-2-Butene	*cis*-2-Butene	Iso-2-butene
1	88.1	69.5	3.9	15.1	11.0	0.5
5	88.3	70.4	4.1	14.5	10.5	0.5
10	82.7	62.2	4.8	18.8	14.0	0.2
15	84.8	65.5	5.1	16.8	12.4	0.2
20	86.3	67.5	5.2	15.6	11.4	0.3

a Reaction condition: *T* = 450 °C, *P* = 0.1 MPa, catalyst weight = 0.5 g, WHSV (E + B) = 1.8 h^−1^, *n*(E)/*n*(B) = 2.

### 3.2 Influence of Si/W ratio on the catalytic performances of W-MCM-41 catalyst

As presented above, stirring time could obviously affect the surface area, dispersion and amount of surface WO_*x*_ species. Definitely, the structure and amount of surface WO_*x*_ species seems to play an important role in determining the catalytic performance. In order to further confirm this effect, W-MCM-41 catalysts were prepared with different Si/W ratio since the varied W loadings may remarkably lead to the changed support structures and the different amount and existing state of WO_*x*_ species.

Table 3 shows the BET surface area, pore volume and pore size of the W-MCM-41 catalysts prepared with different Si/W ratio. It is clear that the sample without the addition of tungstic acid shows the largest BET surface area of up to 1111 m^2^ g^−1^. When the Si/W ratio is 70 and 100, there is no obvious change in the BET surface area but the pore volume slightly increases probably due to the addition of H_2_O_2_ which is used to dissolve tungstic acid and has the effect of the enlargement of pore volume.^31^ The amount of W loading is tested by both XPS and ICP-OES and the results are also listed in Table 3. As characterized by the ICP-OES, the amount of W loading increases from 2.6% to 27.0% as the ratio of Si/W decreases from 100 to 10. The amount of W loading detected by XPS is always lower than the value by ICP-OES. This discrepancy might be resulted from that the result of ICP-OES represents the total amount of W in the catalyst while that of XPS represents the surface amount of W on the catalyst.

**Table 3:** Textural properties of W-MCM-41 catalysts with different Si/W molar ratio

Sample	W (wt%)		BET surface area (m^2^ g^−1^)	Pore volume^a^ (cm^3^ g^−1^)	Pore size^a^ (nm)
	By ICP-OES	By XPS			
MCM-41	0	0	1111	0.53	2.17
W-MCM-41(100)	2.6	2.1	1037	0.61	2.45
W-MCM-41(70)	4.5	3.9	1096	0.62	2.36
W-MCM-41(50)	11.9	6.2	879	0.44	2.38
W-MCM-41(30)	18.1	11.0	654	0.43	2.56
W-MCM-41(10)	27.0	21.0	499	0.43	3.34

a Evaluated by the BJH method.

**Fig. 5:**
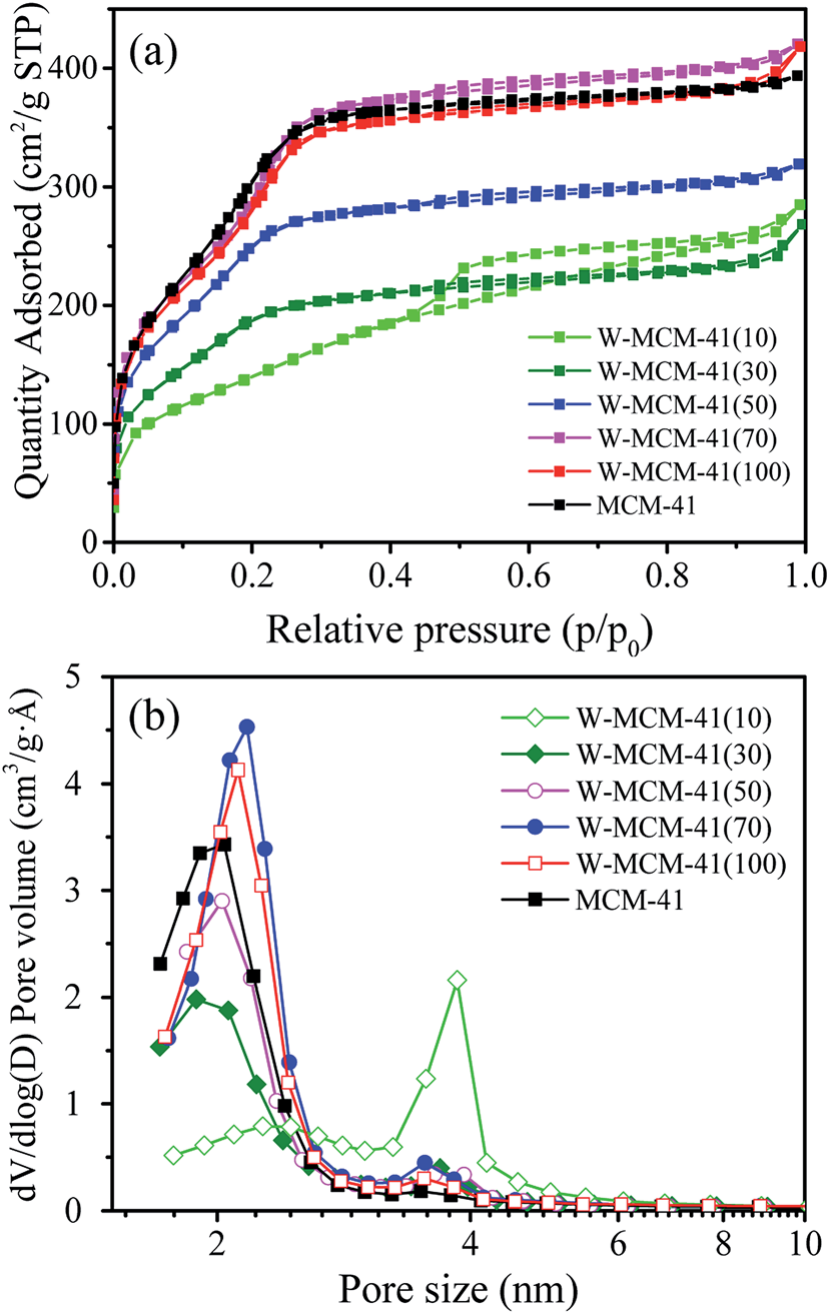
N_2_ adsorption–desorption isotherms (a) and pore size distribution (b) of the W-MCM-41 catalysts with different Si/W molar ratio. The isotherms have been moved verticality to avoid the overlap of isotherms.

As shown in Fig. 5a, all the samples show type IV isotherms with H_1_-hysteresis loops suggesting ordered mesoporous channels.^12^ Especially, for the W-MCM-41(10), the hysteresis loop is much bigger than other samples. As displayed in Fig. 5b, the introduction of W species also increases the pore size of the sample by increasing the amount of pores around 3.5 nm. Over W-MCM-41(10), the amount of pores at about 2 nm is much lower than the other samples while the amount of pores at around 3.5 nm is much higher, which indicates a destroyed zeolite structure over the sample with too much loading of W.

**Fig. 6:**
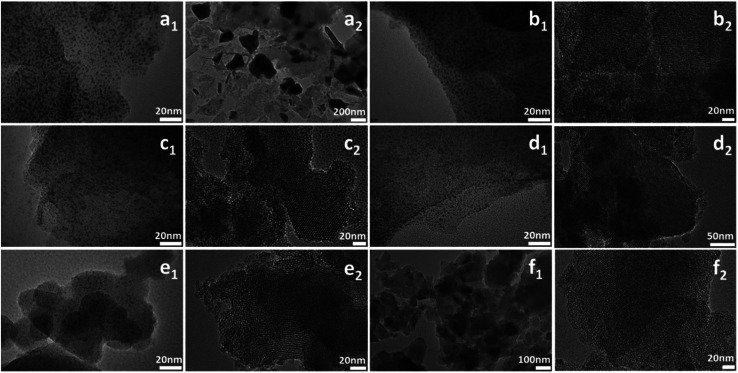
TEM images of (a_1_ and a_2_) W-MCM-41(10), (b_1_ and b_2_) W-MCM-41(30), (c_1_ and c_2_) W-MCM-41(50), (d_1_ and d_2_) W-MCM-41(70), (e_1_ and e_2_) W-MCM-41(100), (f_1_ and f_2_) 18W/MCM-41 catalysts.

TEM images can clearly reflect the morphology of samples. As shown in Fig. 6, all the samples except W-MCM-41(10) show ordered two dimensional pore structures assigned to the MCM-41 zeolite. Moreover, the pore structures of W-MCM-41(30) are not as obvious as the other samples indicating a less ordered pore structures of the sample. The W-MCM-41(10) with the highest content of W shows no regular pore structure and the presence of amorphous SiO_2_. Most of WO_*x*_ species are highly dispersed in the MCM-41 zeolite with a very small particle size of 1–2 nm. However, on the catalyst surface of higher W loading, such as W-MCM-41(10) and W-MCM-41(30) sample, some large WO_*x*_ particles can also be observed. Especially for the W-MCM-41(10), large amount of WO_*x*_ particles is observed and the particle size is over 100 nm. Too much amount of W is indeed destroying the structure of the expected MCM-41 zeolite, which is not suitable for obtaining the well dispersed WO_*x*_ species. For the 18W/MCM-41 sample, the pore structures of MCM-41 is well reserved, but there are only large WO_*x*_ particles unlike the W-MCM-41(30) catalyst showing highly dispersed WO_*x*_ species on the catalyst surface. The one-step prepared method highly increased the dispersion of WO_*x*_ species.

**Fig. 7:**
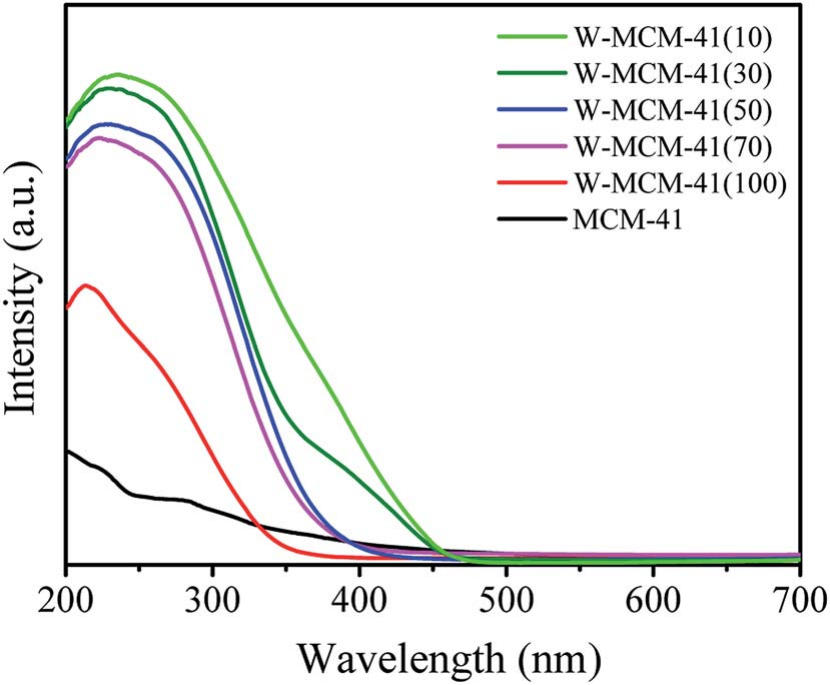
UV-vis DRS spectra of 18W/MCM-41 and W-MCM-41 catalysts with different Si/W molar ratio.

The information about the local molecular coordination and bonding of W species is also detected by UV-vis DR spectra and the results are shown in Fig. 7. The peak intensity at ∼220 nm for W-MCM-41(100) is much stronger than the peak intensity at ∼270 nm, suggesting that most of the W species exist in terms of tetrahedral structure. The amount of octahedral structure at ∼270 nm increases as the increase of the amount of W. Moreover, the peak at ∼400 nm assigned to crystalline WO_3_ appears when the ratio of Si/W is 30 and the peak intensity increases as the Si/W ratio further decreases to 10. It indicates that the WO_*x*_ species are highly dispersed on the catalyst surface except the W-MCM-41(30) and W-MCM-41(10), which is in accordance with the TEM results (Fig. 6).

**Fig. 8:**
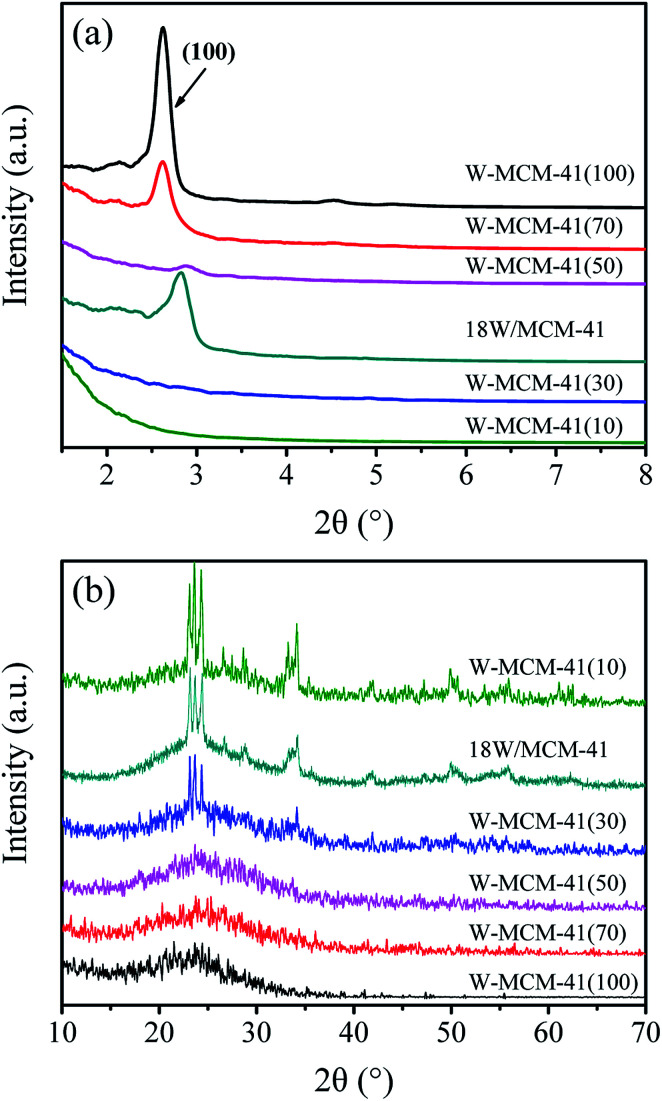
Low-angle XRD patterns (a) and wide-angle XRD patterns (b) of the 18W/MCM-41 and W-MCM-41 catalysts with different Si/W molar ratio.

To further characterize the structure and phase properties, low-angle and wide-angle XRD patterns of these samples are collected and shown in Fig. 8. As shown in Fig. 8a, all the samples except W-MCM-41(10) and W-MCM-41(30) exhibit well-defined (100) reflection indexed on the hexagonal lattice, suggesting the formation of the long-range ordered MCM-41 mesophase in these samples.^36^ Furthermore, the sample with higher Si/W ratio exhibits higher crystallinity than the one with lower Si/W ratio. This may be due to the differences between Si and W atoms in atomic radius (1.32 *versus* 1.41 Å), polarizability and possible coordination valancy.^37^ The W–O–Si likage would distort the vicinal tetrahedral SiO_4_ units. Thus, the high amount of W is detrimental for the formation of long-range ordered pores. Although the (100) reflection of W-MCM-41(30) is not obvious in the XRD patterns, there are still mesopore structures can be observed in the TEM images (Fig. 6b). It indicates that ordered mesopores but not long-range ordered mesopores are existed in this sample.

**Fig. 9:**
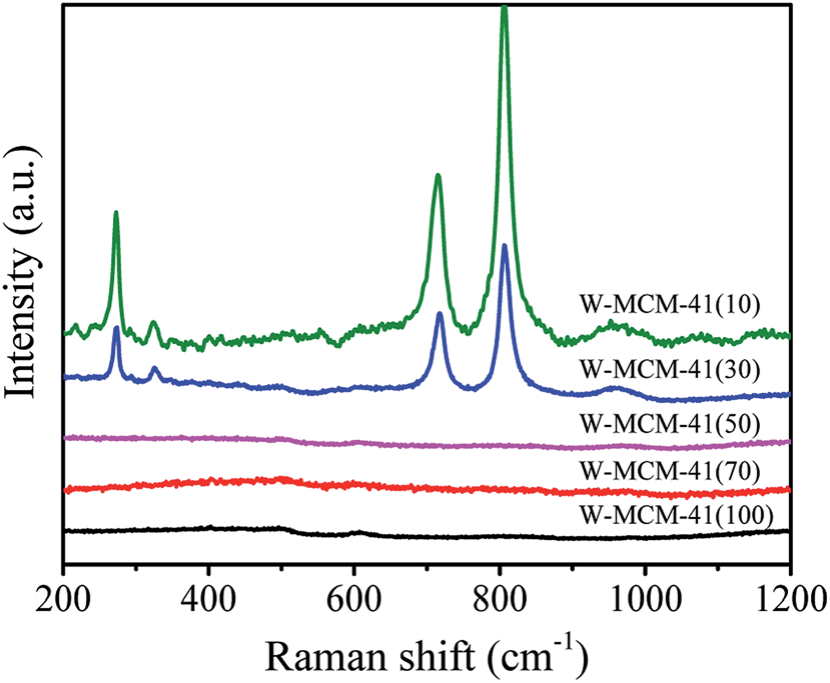
Raman spectra of the W-MCM-41 catalysts with different Si/W molar ratio.

Fig. 8b shows the wide-angle XRD patterns of these samples to reveal the dispersion of WO_*x*_ species. The diffraction peaks assigned to crystal WO_3_ (PDF#72-1465) appear when the Si/W ratio is 30, which are located at 23.1, 23.6, 24.3, 28.9, 33.3 and 34.1°. The intensity of peaks assigned to WO_3_ becomes stronger as the Si/W ratio is 10 indicating the less dispersion of W species on the W-MCM-41(10) catalyst. Moreover, the 18W/MCM-41 catalyst shows more enhanced intensity of WO_3_ compared with W-MCM-41(30) suggesting the poor dispersion of WO_*x*_ species of 18W/MCM-4, which is consistent with the TEM results. The Raman spectra in Fig. 9 also shows that only characteristic peaks assigned to MCM-41 can be observed when the Si/W ratio is higher than 30 suggesting the highly dispersed W species on the support surface. If the Si/W ratio decreases to 30, the peaks located at 274, 705 and 815 cm^−1^ can be observed assigned to the vibration of crystal WO_*x*_, which is the deformation vibration mode of W–O–W, bending vibration mode of W–O, and symmetric stretching vibration mode of W–O, respectively.^38^ These results are well in agreement with the XRD results.

The catalytic performances of these samples are shown in Table 4. It is obvious that both the 1-butene conversion and the propene selectivity increase as the decrease of Si/W ratio from 100 to 30 and then both of them decrease slightly as the further decrease of Si/W ratio from 30 to 10. Therefore, the highest 1-butene conversion of 92.7% and propene selectivity of 80.8% is obtained over W-MCM-41(30) catalyst. Interestingly, the 18W/MCM-41 prepared by the conventional impregnation method with the same loading of W as the W-MCM-41(30) shows notably lower 1-butene conversion of 76.5% and propene selectivity of 34.1%.

**Table 4:** Catalytic performances of W-MCM-41 catalysts with different Si/W molar ratio^a^

Sample	Conversion (%)	Selectivity (%)				
		Propene	C_5+_	*trans*-2-Butene	*cis*-2-Butene	Iso-2-butene
W-MCM-41(100)	59.2	23.0	3.3	39.7	33.8	0.2
W-MCM-41(70)	84.6	63.7	4.5	18.3	13.3	0.2
W-MCM-41(50)	88.3	70.4	4.1	14.5	10.5	0.5
W-MCM-41(30)	92.7	80.8	3.0	8.7	6.3	1.2
18W/MCM-41	76.5	34.1	2.6	35.6	27.3	0.4
W-MCM-41(10)	91.7	78.7	3.2	9.9	7.2	1.0

a Reaction condition: *T* = 450 °C, *P* = 0.1 MPa, catalyst weight = 0.5 g, WHSV (E + B) = 1.8 h^−1^, *n*(E)/*n*(B) = 2.

**Scheme 1:**
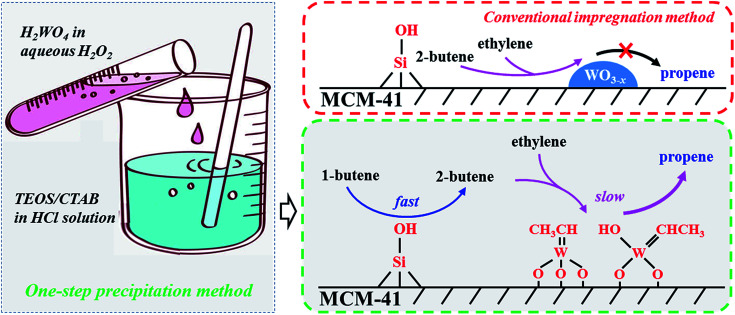
Illustrative drawing of the metathesis reaction of ethylene and 1-butene to propene over W-MCM-41 catalysts prepared by one-step precipitation method and conventional impregnation method.

In general, the metathesis reaction of 1-butene and ethylene to propene evolves three steps including (1) fast isomerization of 1-butene, (2) carbene formation and (3) metathesis reaction. The isomerization is considered to occur on the Si–OH of MCM-41 support which might act as a weak Brønsted acid site for the isomerization of C=C bond (Scheme 1), but lack of the ability of metathesis.^39^ The followed two steps involving the carbene formation and the metathesis reaction take place on the highly dispersed WO_*x*_ species with tetrahedral structure because the crystalline WO_3_ displays a negligible activity for 1-butene isomerization and inactive in metathesis reaction (Scheme 1).^30^ Compared with the first step, the followed two steps are very slow, which is accordance with our catalytic results in Fig. 4. At the very beginning, the fast formed 2-butene can not be immediately converted into propene for the lack of carbene. The carbene formation takes time, which determines the formation of propene. Therefore, at the very beginning, although the 1-butene conversion is high, the propene selectivity is low and it gradually increases with time on stream at the initial one hour (Fig. 4).

For the one step synthesized W-MCM-41 catalysts, most of the WO_*x*_ species are highly dispersed and the higher Si/W ratio corresponds to lower W loading. Although the first isomerization step is not rate-determining for the abundant Si–OH on the MCM-41 support, the followed two steps can be limited for the lack of WO_*x*_ species on the catalyst of higher Si/W ratio. Since the metathesis reaction is a kind of tandem reaction, the suppressing of the second and third steps will hinder the first step to continue. Thus, the conversion of 1-butene should be lower as the W-MCM-41(100) catalyst shows a very lower conversion of 59.2%. It is not hard to understand that the insufficient WO_*x*_ species is also not favorable for the metathesis reaction of 2-butene formed from the first isomerization step and ethylene, which thereby leads to a lower selectivity to metathesis product propene but a higher selectivity to isomerization product, 2-butene. Indeed, the propene selectivity is only 23% and the 2-butene selectivity is higher than 70% over the W-MCM-41(100) catalyst. As the Si/W ratio decreases lower to 10, WO_*x*_ species is prone to form inactive crystal WO_3_ due to the higher W content, smaller surface area and larger pore size of the synthesized amorphous SiO_2_ rather than the highly ordered MCM-41 (Fig. 6, 8, 9 and Table 3). Therefore, both the 1-butene conversion and propene selectivity slightly decrease as the Si/W ratio decreases from 30 to 10. Over the impregnated 18W/MCM-41 catalyst, the dispersion of WO_*x*_ species is much less than the one step preparation method (Scheme 1), which results in less active sites for the second and third step similarly as the case of W/MCM-41(10). It is reasonable that 18W/MCM-41 catalyst exhibits quite lower 1-butene conversion of 76.5% and propene selectivity of 34.1%. In general, the one step precipitation method is an efficient approach to obtain the highly dispersed WO_*x*_ species with desired tetrahedral structure on MCM-41 support, which is responsible for the higher 1-butene conversion and propene selectivity due to the enhanced metathesis reaction of 2-butene and ethylene (Scheme 1).

### 3.3 Influence of upstream isomerization catalysts on the catalyst performances of W-MCM-41 catalyst

Since the metathesis reaction for conversion of 1-butene and ethylene is a kind of the tandem reaction, it is easy to understand that the overall reaction performances can be efficiently improved by accelerating each step in the reactions. Seen from the process of metathesis reaction, the strategy for the higher reaction efficiency can be considered as (1) enhancing the isomerization rate of 1-butene, (2) promoting the conversion of 2-butene and ethylene to propene on the WO_*x*_ species. In the above part, we have intensively discussed the effect of W phase on the reaction performances based on the catalyst characterization and catalytic experiments. Herein, it is planned to improve the catalytic performances by introducing an upstream catalyst onto the W-MCM-41 catalyst as the upstream catalyst can pre-isomerize the 1-butene into 2-butene and thereby propel metathesis reaction. According to this concept, the upstream catalysts are designed as W-MCM-41(30), MgO and SBA-15, respectively, and the corresponding results are listed in Table 5. Among the three upstream catalysts, the MgO shows the highest 1-butene conversion of 93.1% and propene selectivity of 82.7%, while the selectivity to iso-butene slightly decreases. In contrast, SBA-15 catalyst decreases not only the 1-butene conversion but also the propene selectivity. In spite of that the upstream catalyst can be considered to play a role of isomerization and therefore assist the formation of active sites and subsequent 2-butene conversion,^40^ the actual effect on the reaction performances is limited. This result suggests that the isomerization of 1-butene to 2-butene is fast on the metathesis catalyst (Scheme 1), which is not the rate-determining step in the metathesis reaction of 1-butene and ethylene to propene.

**Table 5:** Catalytic performances of W-MCM-41(30) catalysts with different upstream isomerization catalysts^a^

Catalyst loading	Conversion (%)	Selectivity (%)				
		Propene	C_5+_	*trans*-2-Butene	*cis*-2-Butene	Iso-2-butene
No upstream catalyst + W-MCM-41(30)	92.7	80.8	3.0	8.7	6.3	1.2
W-MCM-41(30) + W-MCM-41(30)	93.1	81.3	2.9	8.2	6.0	1.6
MgO + W-MCM-41(30)	93.1	82.7	2.5	8.1	5.9	0.8
SBA-15 + W-MCM-41(30)	91.4	78.0	3.0	10.5	7.5	1.0

a Reaction condition: *T* = 450 °C, *P* = 0.1 MPa, *m*_W-based catalyst_ = 0.5 g, *m*_upstream catalyst_ = 0.5 g, WHSV (E + B) (W-based catalyst) = 1.8 h^−1^, *n*(E)/*n*(B) = 2.

## 4. Conclusion

In conclusion, we have successfully synthesized W-MCM-41 catalysts by a one-step precipitation method and their catalytic performances are tested in the metathesis reaction of 1-butene and ethylene to propene. The results show that both stirring time and Si/W ratio obviously affect the structures of MCM-41 and W phase as confirmed by N_2_ adsorption–desorption, TEM, UV-vis DRS, XRD and Raman characterization. The catalytic evaluation shows that the amount and state of WO_*x*_ species directly determines the 1-butene conversion and propene selectivity as the isolated tetrahedral WO_*x*_ species is mainly responsible for the carbene formation and subsequent metathesis reaction. Moreover, loading an upstream catalyst with the isomerization ability of converting 1-butene to 2-butene could not significantly enhance the reaction performance, which might be due to the fast isomerization of 1-butene to 2-butene and relatively slow metathesis reaction of 2-butene and ethylene to propene on the investigated W-MCM-41 catalysts.

## Conflicts of interest

There are no conflicts to declare.

## Supplementary Material
